# Microstructure and Mechanical Properties of Structural Steels and Alloys

**DOI:** 10.3390/ma16145188

**Published:** 2023-07-24

**Authors:** Anna Bodyakova, Andrey Belyakov

**Affiliations:** Laboratory of Mechanical Properties of Nanostructured Materials and Superalloys, Belgorod State University, 308015 Belgorod, Russia; bodyakova-ai@yandex.ru

## 1. Introduction and Scope

Structural steels and alloys represent a wide domain of materials whose development directly affects human civilization. Mechanical behavior is the principal property of structural steels and alloys. In turn, the mechanical performance of structural steels and alloys depends significantly on their microstructures, phase contents, dislocation substructures, internal stresses, etc. Therefore, studies on the structure–property relationships of steels and alloys are of great practical importance. The development of structural steels and alloys with favorable mechanical properties requires comprehensive investigation of the regularities of microstructure evolution during material processing/manufacturing and various post-processing treatments. The effect of processing regimes/conditions and methods on the microstructures evolved in metals and alloys should be studied in detail to supply materials engineers with deep fundamental and practical knowledge in order to assist the development of advanced structural materials with enhanced mechanical properties. The aim of this Special Issue, “Microstructure and Mechanical Properties of Structural Steels and Alloys”, is to showcase the most recent achievements in theoretical and experimental investigations of microstructures and their effect on mechanical properties of various metallic materials, focusing on scientific breakthroughs in processing and characterization of structural steels and alloys.

## 2. Contributions

This Special Issue presents recent studies dealing with various aspects of microstructure–property relationships of advanced steels and alloys, including commercial and novel materials. A total of 10 papers cover a wide range of structural metals and alloys. The majority of these papers focus on the mechanisms of microstructure evolution and the mechanical properties of metallic materials subjected to various thermo-mechanical, deformation, or heat treatments [1,2,3,4,5,6,7,8]. Readers who are interesting in structural steels may learn from comprehensive investigations of microstructure evolution and its effect on the mechanical properties of diverse steel types [1,3,5,7,8]. Four of these papers [1,3,7,8] present experimental investigations of strengthening mechanisms operating in high-strength steels subjected to various promising treatments including tempforming [1] and quenching–partitioning processing [7]. Some crucial features related to structure–property relationships are detailed for high-strength rebar [3] and a high-speed axle steel [8]. Hydrogen-assisted deformation behavior was clarified by Astafurova et al. [5] for a 316 L austenitic stainless steel. Materials scientists working with aluminum alloys may find interesting a paper investigating changes in microstructure and properties of an Al-Fe alloy subjected to severe plastic deformation [6]. As guest editors, we have the pleasure to note that the present collection of papers is not limited to steels and aluminum alloys. The effect of complex processing techniques involving large strain deformation is studied in two papers, aiming to improve the properties of advanced copper alloys [2,4]. Stress distribution as well as factors affecting stress concentration around a spherical cavity embedded in a cylinder is detailed by Abdelghani et al. [9]. Sun et al. presented a study on particle-reinforced copper–iron composites prepared via powder metallurgy [10].

## 3. Conclusions and Outlook

The papers presented in this Special Issue reflect current research trends in materials science. A great deal of attention is paid for improving material properties by means of microstructure control. The desired microstructures are developed by using various deformation and heat treatments. The key factors affecting the mechanical behavior and fracture of materials are another important matter of research at present. The papers presented in this Special Issue reveal many important features related to structure–property relationships, which significantly expand current knowledge in the field. These papers also disclose topics deserving further investigations and indicate the directions for future research. We believe that materials scientists and other interested readers will find intriguing and useful information in the present Special Issue.

As guest editors, we would like to thank all the authors for their valuable contribution to the present Special Issue. We also express our gratitude to the editorial team of *Materials*, in particular Ms. Karena Tang, for their assistance and support during the preparation of this Special Issue.
